# Initiation of tobacco use during the pandemic: risk factors in the Brazilian context

**DOI:** 10.1590/1980-549720260014

**Published:** 2026-04-27

**Authors:** Deborah Carvalho Malta, Crizian Saar Gomes, Bárbara Aguiar Carrato, Giseli Nogueira Damacena, Paulo Roberto Borges de Souza, Wanessa da Silva Almeida, Célia Landmann Szwarcwald

**Affiliations:** I Universidade Federal de Minas Gerais, School of Nursing - Belo Horizonte (MG), Brazil.; II Universidade Federal de Minas Gerais, School of Medicine - Belo Horizonte (MG), Brazil.; III Fundação Oswaldo Cruz, Institute of Communication and Scientific and Technological Information in Health, Health Information Laboratory - Rio de Janeiro (RJ), Brazil.

**Keywords:** Smokers, COVID-19, Risk Factors, Brazil

## Abstract

**Objective:**

To estimate the incidence of tobacco use during the COVID-19 pandemic among Brazilian adults and to analyze the factors associated with initiation during this period.

**Methods:**

A cross-sectional study using data from the ConVid 2 - Pesquisa de Comportamento (ConVid 2 - Behavior Survey), conducted between July and December 2023 through virtual chain sampling. Prevalence of tobacco use before, during, and after the COVID-19 pandemic, as well as initiation during the pandemic, were assessed. Independent variables included sociodemographic characteristics, health conditions, mental health, and lifestyle factors. Prevalences and 95% confidence intervals (95%CI) were estimated, and factors associated with initiation were investigated using multivariable logistic regression.

**Results:**

The prevalence of smoking was 10.35% before the pandemic, 15.88% during (with an incidence of 5.5% of new smokers), and 12.2% in the post-pandemic period. Higher odds of smoking initiation during the pandemic were observed among individuals not living with a partner (OR=1.44; 95%CI 1.06-1.95), those who self-identified as non-white (OR=2.20; 95%CI 1.17-4.13), those reporting worsening feelings of sadness (OR=1.65; 95%CI 1.11-2.44), and those reporting increased alcohol consumption (OR=6.51; 95%CI 2.89-14.61). Lower odds were found among residents of the Southeast (OR=0.33; 95%CI 0.13-0.79) and Northeast (OR=0.25; 95%CI 0.11-0.57) regions.

**Conclusion:**

These findings highlight the need for public policies targeting more vulnerable populations.

## INTRODUCTION

The COVID-19 pandemic has resulted in more than 700,000 deaths in Brazil and approximately 6.5 million deaths worldwide^1^, with impacts extending beyond the health sector and profoundly affecting social, economic, and political spheres^2^. These effects were unevenly distributed, disproportionately affecting vulnerable populations^3^. Additionally, the pandemic exacerbated physical and mental health problems and led to changes in lifestyle behaviors, including the consumption of alcoholic beverages and tobacco^4^.

Studies examining tobacco use during the pandemic have reported heterogeneous results. Some global studies^4^ indicated an increase in consumption^5^ ^,^ ^6^, whereas research conducted in 19 countries - including the United States^7^, Mexico^8^, France^9^, and China^10^ - did not identify significant changes in the prevalence of daily smokers. A longitudinal study in Pakistan involving approximately 6,000 participants demonstrated contrasting patterns, with some individuals reducing or attempting to quit smoking, while others increased consumption or relapsed^11^.

Most studies focused exclusively on the initial months of the pandemic and did not account for subsequent changes, such as the relaxation of social distancing measures, pandemic fatigue, the introduction of vaccines, and fluctuations in transmission and mortality rates^12^, which may have influenced tobacco use and other health-related behaviors over the course of the pandemic.

Authors have highlighted that stress resulting from epidemics and social distancing measures can generate feelings of fear and insecurity associated with the loss of friends and family, fear of illness, changes in routine, social isolation, and financial losses. These factors may trigger or intensify feelings of sadness, anxiety, depression, and stress^13^ ^,^ ^14^ ^,^ ^15^. In turn, these conditions are associated with an increased urge to smoke^16^ ^,^ ^17^ ^,^ ^18^ ^,^ ^19^ ^,^ ^20^.

In Brazil, the ConVid Comportamentos (ConVid Behaviors) study, conducted by Fundação Oswaldo Cruz (Fiocruz), Universidade Federal de Minas Gerais (UFMG), and Universidade Estadual de Campinas (Unicamp) during the initial months of the pandemic, identified an increase in the intensity of tobacco use among smokers^20^. However, because the study was conducted at the onset of the pandemic, its findings, although indicating an intensification of consumption among habitual smokers, do not allow for the assessment of temporal changes in smoking behavior, nor do they permit verification of a potential increase in smoking initiation in the Brazilian context.

In this context, considering the harmful health effects of smoking, both with regard to the initiation and intensification of this habit during epidemic situations, and the additional risks imposed by the COVID-19 pandemic, it is essential to monitor smoking occurrence and to identify factors associated with its initiation during this period. Systematic monitoring of these indicators is crucial to support public policies aimed at the prevention and control of smoking, as well as to guide health promotion strategies in response to potential behavioral changes arising from health crises.

Thus, the present study aims to estimate the incidence of tobacco use during the COVID-19 pandemic among Brazilian adults and to analyze the factors associated with its initiation during this period.

## METHODS

### Study design

A cross-sectional epidemiological study was conducted using data from ConVid 2 - Pesquisa de Comportamentos (ConVid 2 - Behavioral Research survey), carried out through chain sampling in a virtual environment among the Brazilian adult population from July to December 2023.

The research was conducted by Fiocruz in partnership with UFMG, Unicamp, Universidade Federal de Ouro Preto, and Universidade Federal de Sergipe (UFS).

The target population consisted of individuals aged 18 years old or older who were residing in Brazil during the COVID-19 pandemic.

ConVid 2 addressed topics such as vaccination, COVID-19 and its consequences, economic losses, chronic diseases, and changes in lifestyle and mood, among others^21^.

### Sample

Participant recruitment was carried out using the Respondent Driven Sampling (RDS) method, whose statistical analysis methodology is widely recognized. RDS is considered a complex sampling method, characterized by unequal selection probabilities and clusters composed of individuals recruited by the same participants. To estimate the variances of the indicators of interest, a bootstrap procedure was applied, using multiple simulations of samples generated through the same process that originated the total sample^22^ ^,^ ^23^.

The minimum sample size, estimated at 3,600 individuals aged 18 years or older, was calculated based on a simple random sample, considering a design effect of 2, as suggested by Salganik and Heckathorn^24^. A more detailed description of the use of the RDS method in a virtual environment and of the data collection procedures is available in other publications^25^ ^,^ ^26^.

To ensure the representativeness of the sample with respect to the sociodemographic characteristics of the population, a post-stratification procedure was applied^27^, using as reference the estimates from the 2022 Continuous National Household Sample Survey (Pesquisa Nacional por Amostra de Domicílios Contínua - PNAD), conducted by the Brazilian Institute of Geography and Statistics (Instituto Brasileiro de Geografia e Estatística - IBGE)^28^. The variables considered were gender (male; female), age group (18-39; 40-59; 60 years old or older), educational level (incomplete high school or less; complete high school or incomplete higher education; complete higher education or more), and race/skin color (white; black).

### Variables analyzed

The incidence of tobacco use during the pandemic was assessed using the question: “Before the start of the pandemic, did you smoke any tobacco products?”. Individuals who answered “I had stopped smoking but started smoking again after the onset of the pandemic” or “I did not smoke before; I started smoking after the onset of the pandemic” were classified as new cases during the pandemic.

The following explanatory variables were analyzed in relation to new or incident cases during the pandemic.

#### Sociodemographic and health characteristics

• Gender: male; female;
• Age group: 18-29 years; 30 to 39 years; 40 to 49 years; 50 to 59 years; 60 years old or older;
• Educational level: completed elementary education or incomplete secondary education; completed secondary education or incomplete higher education; completed higher education or higher);
• Race/skin color: White; Black;
• Region of residence: South; Southeast; Central-West; Northeast; and North;
• Lives with a partner: yes; no;
• Health insurance: yes; no;
• Have you ever had COVID-19: yes; no;
• Self-rated health: very good; good; fair; poor; very poor.

#### Mental health

• Sleep problems during the pandemic, based on the question: “Did the pandemic change the quality of your sleep?” A response of “yes” was considered for those who reported “I started having sleep problems” or “I already had sleep problems and they worsened;”
• Anxiety problems during the pandemic, based on the question: “Did the pandemic change your level of anxiety?” A response of “yes” was considered for those who reported “I started having anxiety problems” or “I already had anxiety problems and they worsened;”
• Sadness problems during the pandemic, based on the question: “Did the pandemic change how often you felt sad?” A response of “yes” was considered for those who reported “I started feeling sad with some frequency” or “I already felt sad with some frequency and it worsened.”
• Loneliness problems during the pandemic, based on the question: “Did the pandemic change how often you experienced feelings of loneliness?” A response of “yes” was considered for those who reported “I started experiencing feelings of loneliness with some frequency” or “I already experienced feelings of loneliness with some frequency and they worsened.”

#### Lifestyle factors

• Worsening (increase) in alcohol consumption during the pandemic, based on the question: “Did the pandemic change your consumption of alcoholic beverages?” A response of “yes” was considered for those who reported “Yes, I did not drink before and started drinking after the onset of the pandemic,” “Yes, I had stopped drinking but started again after the onset of the pandemic,” or “Yes, it increased;”
• Worsening (decrease) in the consumption of healthy foods during the pandemic, based on the question: “Did the pandemic change your consumption of healthy foods (fruits and vegetables)?” A response of “yes” was considered for those who reported “It decreased but returned to what it was before the pandemic” or “It decreased and remains so to this day;”
• Worsening (increase) in the consumption of ultra-processed foods during the pandemic, based on the question: “Did the pandemic change your consumption of industrialized products (frozen ready-to-eat meals, sugar-sweetened beverages, soft drinks, packaged snacks, sweets, chocolate, etc.)?” A response of “yes” was considered for those who reported “It increased but returned to what it was before the pandemic” or “It increased and remains so to this day;”
• Worsening (reduction) in physical activity practice during the pandemic, based on the question: “Did the pandemic change the amount of time you spent practicing any type of physical exercise or leisure-time sport?” A response of “yes” was considered for those who reported “It decreased but returned to what it was before the pandemic” or “It decreased and remains so to this day;”
• Worsening (increase) in television viewing time during the pandemic, based on the question: “Did the pandemic change the number of hours you watch television?” A response of “yes” was considered for those who reported “It increased but returned to what it was before” or “It increased and remains so to this day;”
• Worsening (increase) in screen time during the pandemic, based on the question: “Did the pandemic change your time spent using a computer, tablet, or cellphone during leisure time (excluding work and study)?” A response of “yes” was considered for those who reported “It increased but returned to what it was before” or “It increased and remains so to this day.”
• The prevalence of tobacco use before, during, and after the pandemic was also assessed, as described below:
• Post-pandemic prevalence of tobacco use: Individuals were classified as smokers in the post-pandemic period if they answered “I currently smoke, but not daily” or “I smoke daily” to the question: “How often do you smoke any tobacco product? (cigarettes, hand-rolled cigarettes, clove or Bali cigarettes, pipes, cigars, cigarillos, hookah or water pipe, electronic cigarettes, etc. DO NOT consider marijuana cigarettes);”
• Pre-pandemic prevalence of tobacco use: Individuals were classified as smokers before the pandemic if they answered “Yes” to the question: “And before the beginning of the pandemic, did you smoke any tobacco product?”;
• Pandemic-period prevalence of tobacco use: Individuals were classified as smokers during the pandemic if they answered “Yes,” “I had stopped smoking, but started smoking again after the onset of the pandemic,” or “I did not smoke before and started smoking after the onset of the pandemic” to the question: “And before the onset of the pandemic, did you smoke any tobacco product?”.

### Data analysis

The incidence of tobacco use during the pandemic and the prevalence of tobacco use before, during, and after the pandemic were estimated. Logistic regression models were used to assess potential factors associated with the initiation of tobacco use during the pandemic. Variables with a p-value < 0.20 in the univariate analyses were included in the multivariate model. In the final model, variables with a p-value ≤ 0.05 were considered to be associated factors.

All statistical analyses were performed using Stata software, version 17, employing the complex samples module to account for the effects of the sampling design.

### Ethical aspects

The project entitled ConVid2-Pesquisa de Comportamentos was approved by the National Research Ethics Committee on December 22, 2022, under approval number 5.836.202.

## Data availability statement:

The entire dataset that supports the results of this study is available upon request to the author.

## RESULTS

A total of 3,805 individuals were included in the analysis. Most participants were female (52.49%), aged between 18 and 29 years (24.95%), had completed primary education (44.48%), self-identified as Black in terms of race/skin color (56.60%), and resided in the Southeast region (36.20%), as shown in Table 1.

**Table 1.** Characteristics of the study sample. Brazil, Convid Comportamentos, 2023.

Characteristics	% (95%CI)
Gender	
Male	47.51 (42.31-52.77)
Female	52.49 (47.23-57.69)
Age range (years)	
18 to 29	24.95 (19.81-30.90)
30 to 39	20.93 (17.43-24.92)
40 to 49	18.45 (15.91-21.9)
50 to 59	15.58 (12.11-19.83)
60 or more	20.09 (14.71-26.82)
Educational level	
Complete elementary education	44.48 (35.36-53.99)
Complete high school	38.15 (32.34-44.33)
Complete higher education	17.36 (13.61-21.89)
Race/color	
Non-Black	43.40 (39.15-47.74)
Black	56.60 (52.26-60.85)
Region	
South	12.47 (4.77-28.84)
Southeast	36.20 (27.08-46.44)
Central-Weste	8.25 (4.68-14.15)
Northeast	34.97 (23.68-48.25)
North	8.10 (4.97-12.95)

95%CI: 95% Confidence interval.

**Figure 1.**
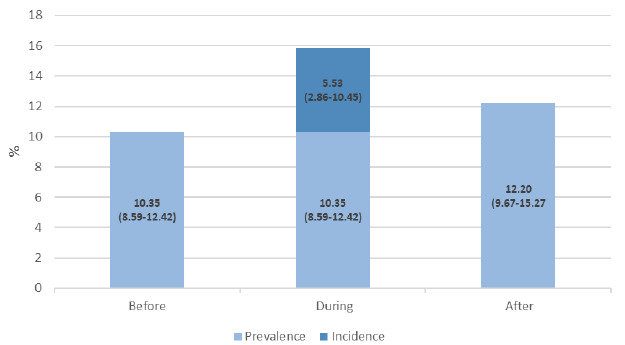
Tobacco use before, during, and after the pandemic. Brazil, Convid Comportamentos, 2023.

Figure 1 illustrates the evolution of tobacco use before, during, and after the pandemic. In the pre-pandemic period, the prevalence was 10.35% (95%CI: 8.59-12.42). During the pandemic, the incidence of smokers was 5.5% (95%CI: 2.86-10.45), resulting in a total prevalence of 15.88% (pre-existing prevalence plus incidence). In the post-pandemic period, the prevalence was 12.2% (95%CI: 9.67-15.27).

Table 2 presents the incidence of tobacco use during the pandemic according to sociodemographic, mental health, and lifestyle characteristics. Higher incidence was observed among individuals aged 18 to 29 years (10.98%), those who self-identified as Black (7.13%), residents of the North region (17.24%), individuals who did not live with a partner (6.79%), and those who reported mental health problems related to the pandemic, such as sleep problems (9.63%), anxiety (8.74%), sadness (9.26%), and loneliness (9.63%). Higher incidence was also identified among participants who reported a worsening of lifestyle behaviors during the pandemic, including increased alcohol consumption (23.65%), decreased consumption of healthy foods (12.84%), increased consumption of ultra-processed foods (12.21%), reduced physical activity (8.31%), and increased screen time (7.93%). No significant differences were observed according to gender, educational level, health insurance coverage, COVID-19 infection, self-rated health status, or increased television viewing time.

**Table 2.** Incidence of tobacco use during the pandemic according to sociodemographic, mental health, and lifestyle characteristics. Brazil, Convid Comportamentos, 2023.

Characteristics	Initiation of tobacco use during the pandemic		
	% (95%CI)	p-value	OR (95%CI)
Gender			
Male	6.25 (2.42-15.18)	0.151	1
Female	4.03 (2.55-6.32)		0.63 (0.33-1.19)
Age range (years)			
18 to 29	10.98 (6.48-17.99)	0.019	1
30 to 39	6.90 (1.92-21.88)		0.60 (0.26-1.36)
40 to 49	2.40 (1.26-4.51)		0.19 (0.07-0.50)
50 to 59	2.33 (1.09-4.90)		0.19 (0.10-0.36)
60 or more	2.66 (0.73-9.18)		0.22 (0.05-0.91)
Educational level			
Elementary	5.90 (1.70-18.47)	0.421	1
Complete high school or incomplete higher education	6.42 (4.64-8.82)		1.09 (0.32-3.62)
Complete higher education or more	2.68 (2.02-3.55)		0.44 (0.11-1.64)
Race/skin color			
Non-Black	3.46 (2.28-5.23)	0.013	1
Black	7.13 (3.26-14.88)		2.14 (1.15-3.96)
Region			
South	9.35 (4.76-15.53)	0.000	1
Southeast	3.85 (2.62-5.63)		0.38 (0.18-0.82)
Central-Weste	8.70 (4.20-17.15)		0.92 (0.55-1.53)
Northeast	3.07 (1.49-6.21)		0.30 (0.15-0.61)
North	17.24 (5.28-43.76)		2.01 (0.92-4.40)
Do you live with a partner?			
Yes	3.65 (1.74-7.50)	0.002	1
No	6.79 (4.07-11.13)		1.92 (1.25-2.95)
Health insurance			
No	5.21 (3.03-8.82)	0.584	1
Yes	6.21 (2.44-14.93)		1.20 (0.61-2.35)
Have you ever had COVID-19?			
Yes	4.32 (2.98-6.22)	0.242	1
No	5.71 (2.33-13.31)		1.33 (0.54-3.28)
I don’t know	8.77 (3.10-22.40)		2.12 (0.76-5.90)
In general, how do you rate your health?			
Very good	4.74 (2.59-8.50)	0.178	1
Good	4.44 (1.93-9.89)		0.93 (0.55-1.56)
Fair	6.82 (3.72-12.18)		1.47 (0.87-2.47)
Poor	9.34 (3.01-25.46)		2.06 (0.92-4.63)
Very poor	10.38 (1.36-49.17)		2.32 (0.25-20.86)
Sleep problems during the pandemic			
No	3.53 (2.07 - 5.92)	0.000	1
Yes	9.63 (4.70 - 18.71)		3.67 (1.67-8.09)
Anxiety problems during the pandemic			
No	3.80 (1.97-7.20)	0.000	1
Yes	8.74 (4.35-16.79)		6.15 (2.88-13.15)
Sadness during the pandemic			
No	3.18 (1.51-6.55)	0.000	1
Yes	9.26 (4.88-16.88)		9.93 (3.89-25.32)
Loneliness during the pandemic			
No	3.82 (1.58-8.95)	0.000	1
Yes	9.63 (5.73-15.74)		6.09 (2.92-12.67)
Worsening (increase) in alcohol consumption during the pandemic			
No	3.09 (2.01-4.72)	0.000	1
Yes	23.65 (9.42-47.98)		14.59 (2.93-72.53)
Worsening (decrease) in consumption of healthy foods during the pandemic			
No	4.06 (2.71-6.04)	0.012	1
Yes	12.84 (4.41-32.0)		1.44 (0.37-5.59)
Worsening (increase) in consumption of ultra-processed foods during the pandemic			
No	4.65 (1.86-11.15)	0.0624	1
Yes	12.21 (7.25-19.83)		0.89 (0.39-2.07)
Worsening (reduction) in physical activity during the pandemic			
No	4.49 (2.31-8.57)	0.000	1
Yes	8.31 (4..24-15.66)		2.39 (1.40-4.06)
Worsening (increase) in TV time during the pandemic			
No	5.53 (2.57-11.52)	0.937	1
Yes	5.67 (3.14-10.05)		2.52 (1.08-5.84)
Worsening (increase) in screen time during the pandemic			
No	3.77 (1.45-9.42)	0.005	1
Yes	7.93 (4.73-13.0)		0.39 (0.04-3.23)

OR: Odds Ratio; 95%: 95% Confidence interval.

In the multivariate model (Table 3), a higher likelihood of initiating tobacco use during the pandemic was observed among individuals who did not live with a partner (OR=1.44; 95%CI: 1.06-1.95), those who self-identified as Black (OR=2.20; 95%CI: 1.17-4.13), those who reported worsening feelings of sadness during the pandemic (OR=1.65; 95%CI: 1.11-2.44), and those who reported increased alcohol consumption during the period (OR=6.51; 95%CI: 2.89-14.61). In contrast, a lower likelihood of initiation was observed with increasing age. Lower odds were also identified among residents of the Southeast (OR=0.33; 95%CI: 0.13-0.79) and Northeast (OR=0.25; 95%CI: 0.11-0.57) regions.

**Table 3.** Factors associated with initiation of tobacco use during the pandemic. Brazil, Convid Comportamentos, 2023.

Characteristics	OR(95%CI)
Age range (years)	
18 to 29	1
30 to 39	0.57 (0.28-1.18)
40 to 49	0.32 (0.14-0.69)
50 to 59	0.37 (0.18-0.77)
60 or more	0.34 (0.12-0.95)
Race/skin color	
Non-Black	1
Black	2.20 (1.17-4.13)
Region	
South	1
Southeast	0.33 (0.13-0.79)
Central-West	0.52 (0.26-1.04)
Northeast	0.25 (0.11-0.57)
North	0.78 (0.38-1.57)
Lives with a partner?	
Yes	1
No	1.44 (1.06-1.95)
Sadness during the pandemic	
No	1
Yes	1.65 (1.11-2.44)
Worsening (increase) in alcohol consumption during the pandemic	
No	1
Yes	6.51 (2.89-14.61)

OR: Odds Ratio; 95%: 95% confidence interval.

## DISCUSSION

The findings of this study indicated an increase in the prevalence of tobacco use in the post-pandemic period, as well as a high incidence during the pandemic. Factors associated with the initiation of tobacco use included not living with a partner, self-identifying as Black, and reporting worsening feelings of sadness and increased alcohol consumption during the pandemic. Conversely, being aged 40 years old or older and residing in the Southeast or Northeast regions of Brazil were identified as protective factors against initiation.

Studies conducted during the pandemic have reported divergent results regarding tobacco consumption, with some indicating an increase^29^ ^,^ ^30^ and others showing stability or a reduction^31^. Research carried out in Italy and the United States identified an increase in smoking prevalence during the pandemic, generally attributed to stressors such as fear, uncertainty related to the disease, and financial difficulties, among others^29^ ^,^ ^30^. The findings of the present study corroborate evidence that the COVID-19 pandemic was associated with an increase in tobacco consumption in Brazil.

In the present study, an association was identified between worsening mental health (reported sadness, anxiety, loneliness, and sleep disturbances) and the initiation of tobacco use. However, in the final model, only reported sadness remained statistically significant due to collinearity among the variables.

Several studies have highlighted the relationship between worsening mental health and tobacco use^19^ ^,^ ^32^ ^,^ ^33^. Similar situations have been described in other catastrophic events, such as Hurricane Katrina in New Orleans, where isolation caused by flooding resulted in psychological distress, reduced social support, and increased socioeconomic vulnerability, factors that led to higher tobacco consumption, which doubled among the Black population^32^.

Evidence suggests that negative emotional states, such as anxiety, depression, and stress, contribute to smoking behavior^19^. Nicotine exerts a direct effect on the central nervous system (CNS), promoting a transient sensation of relief from stress, anguish, and sadness^34^ ^,^ ^35^. This effect occurs primarily through activation of the mesolimbic dopaminergic system, which is responsible for reward and pleasure mechanisms and favors positive reinforcement of smoking behavior. These neurophysiological mechanisms may partially explain the increase in tobacco consumption observed in this study.

Although sleep problems did not remain significant in the final model, some studies have reported this association. Sleep plays a fundamental role in emotional regulation, and sleep deprivation may trigger stress and anxiety^36^. This relationship was also described in studies conducted in Hubei, China, involving approximately 1,000 individuals in social isolation during the COVID-19 pandemic^37^.

The initiation of smoking was shown to be strongly associated with alcohol consumption, representing the variable with the greatest explanatory power. Other studies have also described the co-occurrence of alcohol, tobacco, and other substance use^20^ ^,^ ^38^ ^,^ ^39^. These studies indicate that tobacco use may favor the adoption of other risk behaviors, such as alcohol consumption^40^ ^,^ ^41^, by intensifying the sensation of pleasure resulting from the combination of nicotine and alcohol^39^.

The higher likelihood of initiating tobacco use among younger individuals observed in this study may be associated with the high levels of distress experienced by this age group during the pandemic, resulting from reduced interaction with friends, social isolation, and a lower capacity to cope with stressful situations^20^. Tobacco and other substances may provide temporary relief from stressful circumstances due to the effects of nicotine on the central nervous system^35^.

Regarding the association between not having a partner and a higher likelihood of initiating tobacco use during the pandemic, a study analyzing data from the 2019 National Health Survey also identified a higher prevalence of smoking among individuals without a partner^42^. This finding may be explained by the role of a partner as a source of social support and assistance, factors that contribute to coping with smoking behavior^42^ ^,^ ^43^ ^,^ ^44^.

The North and Southeast regions showed a lower likelihood of initiating tobacco use, possibly reflecting the lower prevalence of smoking observed in states of the Northeast, followed by those in the Southeast region. In both regions, a sustained reduction in tobacco consumption has been observed over recent decades^45^.

It is important to highlight that studies have identified an increase in the use of vaporizers during COVID-19 lockdown periods^46^ ^,^ ^47^. This increase was mainly associated with stress relief, whereas social reasons did not show statistical significance^47^. The present study did not assess the use of new products, such as electronic cigarettes and vapes. However, research conducted with adolescents in Brazil had already identified, in 2019, prior to the pandemic, a rise in the consumption of other tobacco products, such as hookah and electronic cigarettes^48^, as well as increased use of these devices among young adults^45^.

Thus, the increase in smoking prevalence identified in this study may be related not only to behavioral changes resulting from the pandemic, but also to the introduction and popularization of these new products in the country^45^ ^,^ ^48^. The absence of specific questions regarding experimentation with and use of these devices during the pandemic limits a more in-depth analysis of this hypothesis.

The study addresses a highly relevant public health issue and warns of potential setbacks in national tobacco control policies, as well as challenges in achieving tobacco reduction targets^49^. It highlights the strong presence of the tobacco industry on social media, with misleading content and pressure for the adoption of a new regulatory framework that includes the legalization of products such as electronic cigarettes (vapes)^50^, which could negatively influence tobacco use in the country. Therefore, it is essential to strengthen health promotion, education, and regulatory actions, in accordance with the World Health Organization (WHO) Framework Convention on Tobacco Control^51^. It is also necessary to plan specific measures to ensure the continuity of these policies in crisis contexts, such as disasters and pandemics.

This study advances the investigation of cigarette use behavior in Brazil before, during, and after the pandemic by analyzing factors associated with initiation and by innovating through the use of RDS sampling. However, some limitations should be considered, particularly those inherent to internet-based survey sampling. Individuals without internet access have zero probability of being selected, while those with lower levels of education and older adults may experience difficulties in completing the online questionnaire, which can disrupt the development of recruitment networks. Furthermore, because participation is voluntary, selection probabilities and non-response rates cannot be estimated, thereby limiting statistical inference. Another challenge relates to obtaining a representative sample of the Brazilian population. To address this issue, the study applied a post-stratification procedure based on population estimates from the 2022 PNAD, resulting in variable distributions very similar to those observed in PNAD. Nevertheless, the exclusion of variables associated with the outcomes of interest from the post-stratification process may introduce bias into the mean estimates.

Self-reported data are subject to recall bias and misreporting; however, the use of standardized instruments strengthens the analyses and allows for comparability with other studies. Furthermore, the prevalence of tobacco use during the pandemic should be interpreted with caution, as it was not possible to identify individuals who were already smokers and quit during this period.

The study demonstrates that the COVID-19 pandemic intensified smoking in Brazil, with a high incidence during the period and an increase in prevalence after its end. The initiation of tobacco use was associated with individual and contextual factors, with greater vulnerability observed among young people, individuals without a partner, those with worsening emotional health, and those who reported increased alcohol consumption. These findings reinforce the need for policies targeting vulnerable groups, particularly young people and individuals with declining mental health, as well as integrated actions to prevent the use of both alcohol and tobacco.
