# Supplementary material for: Initiation of tobacco use during the pandemic: risk factors in the Brazilian context
Source: Rev Bras Epidemiol. 2026 Apr 27;29:e260014. doi: 10.1590/1980-549720260014 (PMC13123281; doi:10.1590/1980-549720260014)
Supplement: Translation

# Translation: Iniciação ao tabagismo durante a pandemia: fatores de risco no contexto brasileiro

**DOI:** 10.1590/1980-549720260014.2

**Authors:** Deborah Carvalho Malta, Crizian Saar Gomes, Bárbara Aguiar Carrato, Giseli Nogueira Damacena, Paulo Roberto Borges de Souza, Wanessa da Silva Almeida, Célia Landmann Szwarcwald

**Affiliations:** I Universidade Federal de Minas Gerais, Escola de Enfermagem - Belo Horizonte (MG), Brasil.; II Universidade Federal de Minas Gerais, Faculdade de Medicina - Belo Horizonte (MG), Brasil.; III Fundação Oswaldo Cruz, Instituto de Comunicação e Informação Científica e Tecnológica em Saúde, Laboratório de Informação em Saúde - Rio de Janeiro (RJ), Brasil.

**Keywords:** Fumantes, COVID-19, Fatores de Risco, Brasil

## Abstract

**Objetivo:**

Estimar a incidência do uso de tabaco durante a pandemia de COVID-19 em adultos brasileiros e analisar os fatores associados à iniciação nesse período.

**Métodos:**

Estudo transversal com dados da pesquisa ConVid 2 - Pesquisa de Comportamentos, realizada entre julho e dezembro de 2023, conduzida por amostragem em cadeia virtual. Avaliaram-se prevalências de uso de tabaco antes, durante e após a pandemia de COVID-19, bem como a iniciação no período pandêmico. Variáveis independentes incluíram características sociodemográficas, condições de saúde, saúde mental e estilos de vida. Estimaram-se prevalências e IC95%, e fatores associados à iniciação foram investigados por regressão logística multivariada.

**Resultados:**

A prevalência de tabagismo foi de 10,35% antes da pandemia, 15,88% durante (com incidência de 5,5% de novos fumantes) e 12,2% no pós-pandemia. Observou-se maior chance de iniciação durante a pandemia entre pessoas que não viviam com companheiro(a) (OR=1,44; IC95% 1,06-1,95), que se autodeclararam de raça/cor negra (OR=2,20; IC95% 1,17-4,13), aquelas que relataram piora nos sentimentos de tristeza durante a pandemia (OR=1,65; IC95% 1,11-2,44) e entre os que referiram aumento do consumo de bebidas alcóolicas (OR=6,51; IC95% 2,89-14,61). Menores chances foram observadas entre os residentes nas Regiões Sudeste (OR=0,33; IC95% 0,13-0,79) e Nordeste (OR=0,25; IC95% 0,11-0,57).

**Conclusão:**

Esses achados reforçam a necessidade de políticas públicas voltadas a populações mais vulneráveis.

## INTRODUÇÃO

A pandemia de COVID-19 causou mais de 700 mil mortes no Brasil e 6,5 milhões no mundo^1^, com impactos que ultrapassaram a saúde, afetando profundamente as esferas social, econômica e política^2^. Esses efeitos foram desiguais, atingindo mais intensamente populações vulneráveis^3^. Além disso, a pandemia agravou a saúde física e mental e provocou mudanças nos estilos de vida, incluindo o consumo de bebidas alcóolicas e tabaco^4^.

Estudos sobre o uso de tabaco durante a pandemia apresentam resultados heterogêneos. Alguns trabalhos globais apontaram aumento do consumo^5^ ^,^ ^6^, enquanto pesquisas em 19 países - como Estados Unidos^7^, México^8^, França^9^ e China^10^ - não identificaram mudanças significativas na prevalência de fumantes diários. Um estudo longitudinal no Paquistão, com cerca de 6.000 participantes, mostrou efeitos opostos: parte reduziu ou tentou cessar o tabagismo, enquanto outra parte aumentou o consumo ou teve recaídas^11^.

A maioria dos estudos analisou apenas os meses iniciais da pandemia, não contemplando as mudanças que ocorreram posteriormente, como flexibilização do distanciamento, fadiga pandêmica, chegada das vacinas e variações da transmissão e da mortalidade^12^, que podem ter alterado o uso do tabaco e de outros comportamentos de saúde ao longo da pandemia.

Autores destacam que o estresse decorrente de epidemias e das medidas de distanciamento social pode gerar sentimentos de medo e insegurança, associados à perda de amigos e familiares, ao receio da doença, às mudanças na rotina, ao isolamento social e às perdas financeiras. Esses fatores podem desencadear ou intensificar quadros de tristeza, ansiedade, depressão e estresse^13^ ^,^ ^14^ ^,^ ^15^. Tais condições, por sua vez, estão associadas ao aumento do desejo de fumar^16^ ^,^ ^17^ ^,^ ^18^ ^,^ ^19^ ^,^ ^20^.

No Brasil, o estudo “ConVid Comportamentos”, conduzido pela Fundação Oswaldo Cruz (Fiocruz), pela Universidade Federal de Minas Gerais (UFMG) e pela Universidade Estadual de Campinas (Unicamp) nos primeiros meses da pandemia, identificou o aumento da intensidade do uso de tabaco entre fumantes^20^. Entretanto, por ter sido realizado ainda no início da pandemia, seus achados, embora indiquem uma intensificação do consumo entre fumantes habituais, não permitem avaliar mudanças temporais no comportamento de fumar, tampouco verificar se houve aumento na iniciação ao tabagismo no contexto brasileiro.

Nesse cenário, considerando os efeitos nocivos do tabagismo para a saúde, tanto em relação à iniciação quanto à intensificação desse hábito em situações epidêmicas, e os riscos adicionais impostos pela pandemia de COVID-19, torna-se fundamental monitorar a ocorrência do tabagismo e identificar os fatores associados à sua iniciação durante esse período. O acompanhamento sistemático desses indicadores é essencial para subsidiar políticas públicas de prevenção e controle do tabagismo, bem como para orientar estratégias de promoção da saúde diante de possíveis mudanças comportamentais decorrentes de crises sanitárias.

Assim, o presente estudo tem como objetivo estimar a incidência do uso de tabaco durante a pandemia de COVID-19 em adultos brasileiros e analisar os fatores associados à iniciação nesse período.

## MÉTODOS

### Desenho do estudo

Estudo epidemiológico transversal com dados da pesquisa “ConVid 2 - Pesquisa de Comportamentos”, realizada por amostragem em cadeia em ambiente virtual, com a população adulta brasileira, no período de julho a dezembro de 2023.

A pesquisa foi conduzida pela Fiocruz, em parceria com a UFMG, a Unicamp, a Universidade Federal de Ouro Preto e a Universidade Federal de Sergipe (UFS).

A população-alvo incluiu indivíduos com 18 anos ou mais de idade, residentes no Brasil durante a pandemia de COVID-19.

A ConVid 2 abordou temas como vacinação, COVID-19 e suas consequências, perdas econômicas, doenças crônicas, alterações nos estilos de vida e no estado de ânimo, entre outros^21^.

### Amostra

O recrutamento dos participantes foi realizado por meio do método Respondent Driven Sampling (RDS), cuja metodologia de análise estatística é amplamente reconhecida. O RDS é considerado um método de amostragem complexo, caracterizado por probabilidades desiguais de seleção e conglomerados compostos por pessoas recrutadas pelos mesmos participantes. Para a estimativa das variâncias dos indicadores de interesse, foi utilizado o procedimento de bootstrap, por meio de múltiplas simulações de amostras geradas com o mesmo processo que originou a amostra total^22^ ^,^ ^23^.

O cálculo do tamanho mínimo da amostra, estimado em 3.600 indivíduos com 18 anos ou mais, foi realizado com base em uma amostra aleatória simples, considerando-se um efeito de desenho igual a 2, conforme sugerido por Salganik e Heckathorn^24^. Um detalhamento mais aprofundado sobre a utilização do método RDS em ambiente virtual e sobre o procedimento de coleta de informações está disponível em outra publicação^25^ ^,^ ^26^.

Para garantir a representatividade da amostra em relação às características sociodemográficas da população, foi aplicado um procedimento de pós-estratificação^27^, utilizando como referência as estimativas da Pesquisa Nacional por Amostra de Domicílios Contínua (PNAD) 2022, do Instituto Brasileiro de Geografia e Estatística (IBGE)^28^. As variáveis consideradas foram: sexo (masculino; feminino), faixa etária (18-39; 40-59; 60 anos ou mais), grau de escolaridade (ensino médio incompleto ou menos; ensino médio completo ou superior incompleto; superior completo ou mais) e raça/cor da pele (branca; negra).

### Variáveis analisadas

A incidência do uso de tabaco durante a pandemia foi analisada por meio da pergunta: “Antes do início da pandemia, você fumava algum produto do tabaco?”. Foram classificados como novos casos durante a pandemia os indivíduos que responderam “Tinha parado de fumar, mas comecei a fumar novamente após o início da pandemia” ou “Não fumava antes, comecei a fumar após o início da pandemia”.

Foram analisadas as seguintes variáveis explicativas para os casos novos ou incidentes durante a pandemia.

#### Características sociodemográficas e de saúde

• Sexo: masculino; feminino;
• Faixa etária: 18-29 anos; 30 a 39 anos; 40 a 49 anos; 50-59 anos; 60 anos ou mais;
• Escolaridade: fundamental completo ou médio incompleto; médio completo ou superior incompleto; superior completo ou mais);
• Raça/cor da pele: branca; negra;
• Região de moradia: Sul; Sudeste; Centro-Oeste, Nordeste e Norte;
• Vive com cônjuge: sim; não;
• Plano de saúde: sim; não;
• Alguma vez você teve COVID-19: sim; não;
• Autoavaliação de saúde: muito boa; boa; regular; ruim; muito ruim.

#### Saúde mental

• Problemas de sono durante a pandemia, segundo a pergunta: “A pandemia modificou a qualidade do seu sono?”. Considerou-se como resposta “sim” aqueles que afirmaram “Comecei a ter problemas de sono” ou “Eu já tinha problemas de sono e eles pioraram”;
• Problemas de ansiedade durante a pandemia, segundo a pergunta: “A pandemia modificou o seu estado de ansiedade?”. Considerou-se como resposta “sim” aqueles que afirmaram “Comecei a ter problemas de ansiedade” ou “Eu já tinha problemas de ansiedade e eles pioraram”;
• Problemas de tristeza durante a pandemia, segundo a pergunta: “A pandemia modificou a frequência com que você se sentiu triste?”. Considerou-se como resposta “sim” aqueles que afirmaram: “Comecei a ter sentimentos de tristeza com alguma frequência” ou “Eu já tinha sentimentos de tristeza com alguma frequência e eles pioraram”.
• Problemas de solidão durante a pandemia, segundo a pergunta: “A pandemia modificou a frequência com que você teve sentimentos de solidão?”. Considerou-se como resposta “sim” aqueles que afirmaram “Comecei a ter sentimentos de solidão com alguma frequência” ou “Eu já tinha sentimentos de solidão com alguma frequência e eles pioraram”.

#### Estilos de vida

• Piora (aumento) do consumo de bebidas alcoólicas durante a pandemia, segundo a pergunta: “A pandemia modificou o seu consumo de bebidas alcoólicas?”. Considerou-se como resposta “sim” aqueles que afirmaram “Sim, não bebia antes e comecei a beber após o início da pandemia”, “Sim, tinha parado de beber, mas comecei a beber novamente após o início da pandemia” ou “Sim, aumentou”;
• Piora (diminuição) do consumo de alimentos saudáveis durante a pandemia, segundo a pergunta: “A pandemia modificou o seu consumo de alimentos saudáveis (frutas, verduras e legumes)?”. Considerou-se como resposta “sim” aqueles que afirmaram “Diminuiu, mas voltou ao que era antes da pandemia” ou “Diminuiu e permanece assim até hoje”;
• Piora (aumento) do consumo de alimentos ultraprocessados durante a pandemia, segundo a pergunta: “A pandemia modificou o seu consumo de produtos industrializados (comidas prontas congeladas, bebidas açucaradas, refrigerantes, salgadinhos de pacote, doces, chocolate etc.)?”. Considerou-se como resposta “sim” aqueles que afirmaram “Aumentou, mas voltou ao que era antes da pandemia” ou “Aumentou e permanece assim até hoje”;
• Piora (redução) da prática de atividade física durante a pandemia, segundo a pergunta: “A pandemia modificou o seu tempo de prática de algum tipo de exercício físico ou esporte no lazer?”. Considerou-se como resposta “sim” aqueles que afirmaram “Diminuiu, mas voltou ao que era antes da pandemia” ou “Diminuiu e permanece assim até hoje”;
• Piora (aumento) do tempo de TV durante a pandemia, segundo a pergunta: “A pandemia modificou o número de horas que você assiste televisão?”. Considerou-se como resposta “sim” aqueles que afirmaram “Aumentou, mas voltou ao que era antes” ou “Aumentou e permanece assim até hoje”;
• Piora (aumento) do tempo de telas durante a pandemia, segundo a pergunta: “A pandemia modificou o seu tempo de uso de computador, tablet ou celular no tempo livre (excluindo trabalho e estudo)?”. Considerou-se como resposta “sim” aqueles que afirmaram “Aumentou, mas voltou ao que era antes” ou “Aumentou e permanece assim até hoje”.
• Também foram avaliadas as prevalências de uso de tabaco antes, durante e após a pandemia, conforme descrito a seguir:
• Prevalência de uso de tabaco no pós-pandemia: Foram classificados como fumantes no pós-pandemia os indivíduos que responderam “Fumo atualmente, mas não diariamente” ou “Fumo diariamente” à pergunta: “Com que frequência você fuma algum produto do tabaco? (cigarros, cigarros de palha ou enrolados à mão, cigarros de cravo ou de Bali, cachimbos, cigarrilhas, narguilé ou cachimbo d’água, cigarro eletrônico etc. NÃO considere cigarros de maconha).”;
• Prevalência do uso de tabaco antes da pandemia: Foram considerados como fumantes antes da pandemia os que responderam “Sim” à pergunta: “E antes do início da pandemia, você fumava algum produto do tabaco?”;
• Prevalência do uso de tabaco durante a pandemia: Foram considerados fumantes durante a pandemia os indivíduos que responderam “Sim”, “Tinha parado de fumar, mas comecei a fumar novamente após o início da pandemia” ou “Não fumava antes, comecei a fumar após o início da pandemia” à pergunta “E antes do início da pandemia, você fumava algum produto do tabaco?”.

### Análise dos dados

Foram estimadas a incidência de uso do tabaco durante a pandemia e as prevalências do uso de tabaco antes, durante e após a pandemia. Para verificar os possíveis fatores associados à iniciação do uso de tabaco durante a pandemia, utilizaram-se modelos de regressão logística. Foram incluídas no modelo multivariado as variáveis que apresentaram valor de p<0,20 nas análises univariadas. No modelo final, consideraram-se como fatores associados as variáveis com valor de p≤0,05.

Todas as análises estatísticas foram realizadas no software Stata, versão 17, utilizando o módulo de amostras complexas, de modo a considerar os efeitos do plano de amostragem.

### Aspectos éticos

O projeto denominado ConVid2-Pesquisa de Comportamentos foi aprovado pela Comissão Nacional de Ética em Pesquisa em 22 de dezembro de 2022, sob o nº 5.836.202.

## Declaração de disponibilidade de dados:

Todo o conjunto de dados que dá suporte aos resultados deste estudo está disponível mediante solicitação ao autor correspondente.

## RESULTADOS

Foram analisados 3.805 indivíduos. A maioria era do sexo feminino (52,49%), tinha entre 18 e 29 anos de idade (24,95%), apresentava ensino fundamental completo (44,48%), era de raça/cor negra (56,60%) e residia na região Sudeste (36,20%), conforme Tabela 1.

**Tabela 1.** Características da amostra estudada. Brasil, Convid Comportamentos, 2023.

Variáveis	% (IC95%)
Sexo	
Masculino	47,51 (42,31-52,77)
Feminino	52,49 (47,23-57,69)
Faixa etária (anos)	
18 a 29	24,95 (19,81-30,90)
30 a 39	20,93 (17,43-24,92)
40 a 49	18,45 (15,91-21,9)
50 a 59	15,58 (12,11-19,83)
60 ou mais	20,09 (14,71-26,82)
Escolaridade	
Fundamental completo	44,48 (35,36-53,99)
Médio completo	38,15 (32,34-44,33)
Superior completo	17,36 (13,61-21,89)
Raça/cor	
Não negro	43,40 (39,15-47,74)
Negro	56,60 (52,26-60,85)
Região	
Sul	12,47 (4,77-28,84)
Sudeste	36,20 (27,08-46,44)
Centro-Oeste	8,25 (4,68-14,15)
Nordeste	34,97 (23,68-48,25)
Norte	8,10 (4,97-12,95)

IC95%: Intervalo de confiança de 95%.

A Figura 1 apresenta a evolução do uso de tabaco antes, durante e após a pandemia. Observa-se que, no período pré-pandemia, a prevalência foi de 10,35% (IC95% 8,59-12,42). Durante a pandemia, a incidência de fumantes foi de 5,5% (IC95% 2,86-10,45), resultando em uma prevalência total de 15,88% (prevalência pré-existente somada à incidência). Já no período pós-pandemia, a prevalência foi de 12,2% (IC95% 9,67-15,27).

**Figura 1.** Uso de tabaco antes, durante e após a pandemia. Brasil, Convid Comportamentos, 2023.

A Tabela 2 apresenta a incidência do uso de tabaco durante a pandemia, segundo características sociodemográficas, de saúde mental e de estilo de vida. Observa-se maior uso de tabaco entre indivíduos de 18 a 29 anos (10,98%), entre aqueles que se autodeclararam de raça/cor negra (7,13%), residentes na região Norte (17,24%), pessoas que não viviam com companheiro(a) (6,79%) e entre os que relataram problemas de saúde mental decorrentes da pandemia, como problemas de sono (9,63%), ansiedade (8,74%), tristeza (9,26%) e solidão (9,63%). Também foi observado maior uso entre aqueles que referiram piora nos estilos de vida durante a pandemia, incluindo aumento do consumo de bebidas alcoólicas (23,65%), diminuição do consumo de alimentos saudáveis (12,84%), aumento do consumo de ultraprocessados (12,21%), redução da prática de atividade física (8,31%) e aumento do tempo de telas (7,93%). Não foram observadas diferenças significativas segundo sexo, escolaridade, posse de plano de saúde, infecção por COVID-19, autoavaliação de saúde ou aumento do tempo de TV.

**Tabela 2.** Incidência do uso de tabaco durante a pandemia segundo características sociodemográficas, de saúde mental e estilo de vida. Brasil, Convid Comportamentos, 2023.

Variáveis	Iniciação do uso de tabaco durante a pandemia		
	% (IC95%)	Valor p	OR (IC95%)
Sexo			
Masculino	6,25 (2,42-15,18)	0,151	1
Feminino	4,03 (2,55-6,32)		0,63 (0,33-1,19)
Faixa etária (anos)			
18 a 29	10,98 (6,48-17,99)	0,019	1
30 a 39	6,90 (1,92-21,88)		0,60 (0,26-1,36)
40 a 49	2,40 (1,26-4,51)		0,19 (0,07-0,50)
50 a 59	2,33 (1,09-4,90)		0,19 (0,10-0,36)
60 ou mais	2,66 (0,73-9,18)		0,22 (0,05-0,91)
Escolaridade			
Fundamental	5,90 (1,70-18,47)	0,421	1
Médio completo ou superior incompleto	6,42 (4,64-8,82)		1,09 (0,32-3,62)
Superior completo ou mais	2,68 (2,02-3,55)		0,44 (0,11-1,64)
Raça/cor			
Não negro	3,46 (2,28-5,23)	0,013	1
Negro	7,13 (3,26-14,88)		2,14 (1,15-3,96)
Região			
Sul	9,35 (4,76-15,53)	0,000	1
Sudeste	3,85 (2,62-5,63)		0,38 (0,18-0,82)
Centro-Oeste	8,70 (4,20-17,15)		0,92 (0,55-1,53)
Nordeste	3,07 (1,49-6,21)		0,30 (0,15-0,61)
Norte	17,24 (5,28-43,76)		2,01 (0,92-4,40)
Vive com cônjuge ou companheiro(a)?			
Sim	3,65 (1,74-7,50)	0,002	1
Não	6,79 (4,07-11,13)		1,92 (1,25-2,95)
Plano de saúde			
Não	5,21 (3,03-8,82)	0,584	1
Sim	6,21 (2,44-14,93)		1,20 (0,61-2,35)
Alguma vez você teve COVID-19?			
Sim	4,32 (2,98-6,22)	0,242	1
Não	5,71 (2,33-13,31)		1,33 (0,54-3,28)
Não sei	8,77 (3,10-22,40)		2,12 (0,76-5,90)
Em geral, como você avalia a sua saúde?			
Muito boa	4,74 (2,59-8,50)	0,178	1
Boa	4,44 (1,93-9,89)		0,93 (0,55-1,56)
Regular	6,82 (3,72-12,18)		1,47 (0,87-2,47)
Ruim	9,34 (3,01-25,46)		2,06 (0,92-4,63)
Muito ruim	10,38 (1,36-49,17)		2,32 (0,25-20,86)
Problemas de sono durante a pandemia			
Não	3,53 (2,07 - 5,92)	0,000	1
Sim	9,63 (4,70 - 18,71)		3,67 (1,67-8,09)
Problemas de ansiedade durante a pandemia			
Não	3,80 (1,97-7,20)	0,000	1
Sim	8,74 (4,35-16,79)		6,15 (2,88-13,15)
Problemas de tristeza durante a pandemia			
Não	3,18 (1,51-6,55)	0,000	1
Sim	9,26 (4,88-16,88)		9,93 (3,89-25,32)
Problemas de solidão durante a pandemia			
Não	3,82 (1,58-8,95)	0,000	1
Sim	9,63 (5,73-15,74)		6,09 (2,92-12,67)
Piora (aumento) do consumo de bebidas alcóolicas durante a pandemia			
Não	3,09 (2,01-4,72)	0,000	1
Sim	23,65 (9,42-47,98)		14,59 (2,93-72,53)
Piora (diminuição) do consumo de alimentos saudáveis durante a pandemia			
Não	4,06 (2,71-6,04)	0,012	1
Sim	12,84 (4,41-32,0)		1,44 (0,37-5,59)
Piora (aumento) do consumo de alimentos ultraprocessados durante a pandemia			
Não	4,65 (1,86-11,15)	0,0624	1
Sim	12,21 (7,25-19,83)		0,89 (0,39-2,07)
Piora (redução) da prática de atividade física durante a pandemia			
Não	4,49 (2,31-8,57)	0,000	1
Sim	8,31 (4.,24-15,66)		2,39 (1,40-4,06)
Piora (aumento) do tempo de TV durante a pandemia			
Não	5,53 (2,57-11,52)	0,937	1
Sim	5,67 (3,14-10.05)		2,52 (1,08-5,84)
Piora (aumento) do tempo de telas durante a pandemia			
Não	3,77 (1,45-9,42)	0,005	1
Sim	7,93 (4,73-13,0)		0,39 (0,04-3,23)

OR: Odds Ratio; IC95%: Intervalo de confiança de 95%.

No modelo multivariado (Tabela 3), observou-se maior chance de iniciação do uso de tabaco durante a pandemia entre pessoas que não viviam com companheiro(a) (OR=1,44; IC95% 1,06-1,95), os que se autodeclararam de raça/cor negra (OR=2,20; IC95% 1,17-4,13), aquelas que relataram piora nos sentimentos de tristeza durante a pandemia (OR=1,65; IC95% 1,11-2,44) e entre os que referiram aumento do consumo de bebidas alcóolicas no período (OR=6,51; IC95% 2,89-14,61). Por outro lado, verificou-se menor chance de iniciação com o aumento da faixa etária. Menores chances também foram observadas entre residentes nas regiões Sudeste (OR=0,33; IC95% 0,13-0,79) e Nordeste (OR=0,25; IC95% 0,11-0,57).

**Tabela 3.** Fatores associados à iniciação do uso de tabaco durante a pandemia. Brasil, Convid Comportamentos, 2023.

Variáveis	OR(IC95%)
Faixa etária (anos)	
18 a 29	1
30 a 39	0,57 (0,28-1,18)
40 a 49	0,32 (0,14-0,69)
50 a 59	0,37 (0,18-0,77)
60 ou mais	0,34 (0,12-0,95)
Raça/cor	
Não negro	1
Negro	2,20 (1,17-4,13)
Região	
Sul	1
Sudeste	0,33 (0,13-0,79)
Centro-Oeste	0,52 (0,26-1,04)
Nordeste	0,25 (0,11-0,57)
Norte	0,78 (0,38-1,57)
Vive com cônjuge ou companheiro(a)?	
Sim	1
Não	1,44 (1,06-1,95)
Problemas de tristeza durante a pandemia	
Não	1
Sim	1,65 (1,11-2,44)
Piora (aumento) do consumo de bebidas alcóolicas durante a pandemia	
Não	1
Sim	6,51 (2,89-14,61)

OR: Odds Ratio; IC95%: Intervalo de confiança de 95%.

## DISCUSSÃO

Os achados deste estudo evidenciaram aumento da prevalência do uso de tabaco no período pós-pandemia, assim como alta incidência durante a pandemia. Os fatores associados à iniciação do consumo de tabaco foram: não viver com companheiro(a), autodeclarar raça/cor negra e relatar piora dos sentimentos de tristeza e aumento do consumo de bebidas alcóolicas durante a pandemia. Por outro lado, ter idade acima de 40 anos e residir nas regiões Sudeste ou Nordeste mostraram-se fatores de proteção contra a iniciação.

Estudos conduzidos durante a pandemia apontaram resultados distintos sobre o consumo de tabaco, com alguns indicando aumento^29^ ^,^ ^30^ e outros relatando estabilidade ou redução^31^. Pesquisas realizadas na Itália e nos Estados Unidos mostraram aumento da prevalência de tabagismo na população durante a pandemia, geralmente atribuído a fatores estressores, como medo, incerteza sobre a doença, dificuldades financeiras, entre outros^29^ ^,^ ^30^. Os achados do presente estudo confirmam que a pandemia de COVID-19 resultou em aumento do consumo de tabaco no Brasil.

No presente estudo, foi identificada associação entre a piora da saúde mental (relatos de tristeza, ansiedade, solidão e piora do sono) e a iniciação do uso do tabaco. Contudo, no modelo final, apenas o relato de tristeza permaneceu significativo, em razão da colinearidade entre as variáveis.

Diversos estudos apontam a relação entre a piora da saúde mental e o uso de tabaco^19^ ^,^ ^32^ ^,^ ^33^. Situações semelhantes já foram descritas em outras catástrofes, como no furacão Katrina, em Nova Orleans, em que o isolamento causado pelos alagamentos gerou sofrimento mental, baixo apoio social e maior vulnerabilidade socioeconômica, fatores que levaram ao aumento do consumo de tabaco, chegando a dobrar entre a população negra^32^.

Evidências apontam que estados emocionais negativos, como ansiedade, depressão e estresse, são fatores contribuintes para o tabagismo^19^. A nicotina exerce ação direta sobre o Sistema Nervoso Central (SNC), promovendo sensação transitória de alívio do estresse, da angústia e da tristeza^34^ ^,^ ^35^. Esse efeito ocorre principalmente pela ativação do sistema dopaminérgico mesolímbico, responsável pelos mecanismos de recompensa e prazer, favorecendo o reforço positivo do comportamento de fumar. Tais alterações neurofisiológicas podem explicar, em parte, o aumento do consumo de tabaco observado neste estudo.

Embora os problemas de sono não tenham se mantido no modelo final, alguns estudos apontam essa associação. O sono desempenha um papel fundamental na regulação emocional, e sua perda pode desencadear situações de estresse e ansiedade^36^. Essa relação também foi descrita em estudos realizados em Hubei, na China, com aproximadamente 1.000 indivíduos em isolamento social durante a pandemia de COVID-19^37^.

A iniciação ao tabagismo mostrou-se fortemente associada ao consumo de bebidas alcoólicas, sendo a variável com maior poder explicativo. Outros estudos também descrevem a coocorrência de consumo de bebidas alcóolicas, tabaco e outras substâncias^20^ ^,^ ^38^ ^,^ ^39^. Esses estudos indicam que o tabaco pode favorecer a adoção de outros comportamentos de risco, como o consumo de bebidas alcoólicas^40^ ^,^ ^41^, intensificando a sensação de prazer devido à combinação da nicotina com o álcool^39^.

A maior chance de iniciação do consumo de tabaco entre jovens, observada no presente estudo, pode estar associada ao elevado sofrimento experimentado por essa faixa etária durante a pandemia, decorrente da falta de interação com amigos, do isolamento social e da menor capacidade de lidar com situações estressoras^20^. O tabaco e outras substâncias podem proporcionar alívio temporário diante de situações estressoras, devido à ação da nicotina sobre o sistema nervoso central^35^.

No que se refere à associação entre não possuir companheiro(a) e a maior chance de iniciação do uso de tabaco durante a pandemia, um estudo que analisou dados da Pesquisa Nacional de Saúde 2019 também encontrou maior prevalência de tabagismo entre indivíduos que não tinham companheiro(a)^42^. Esse achado pode ser explicado pelo papel do(a) companheiro(a) como fonte de apoio e suporte social, fatores que contribuem para o enfrentamento do tabagismo^42^ ^,^ ^43^ ^,^ ^44^.

As regiões Norte e Sudeste apresentaram menor chance de iniciação ao uso do tabaco, possivelmente devido às menores prevalências de tabagismo nos estados do Nordeste, seguidos pelos estados da região Sudeste. Em ambas as regiões, a redução do consumo de tabaco tem sido constante nas últimas décadas^45^.

É importante destacar que estudos identificaram que o uso de vaporizadores aumentou durante os períodos de lockdown da COVID-19^46^ ^,^ ^47^. Esse aumento esteve associado principalmente ao alívio do estresse, enquanto os motivos de ordem social não apresentaram significância estatística^47^. O presente estudo não avaliou o uso de novos produtos, como cigarros eletrônicos e vapes. Entretanto, pesquisas realizadas com adolescentes no Brasil já haviam identificado, em 2019, antes da pandemia, o crescimento do consumo de outros produtos derivados do tabaco, como narguilé e cigarros eletrônicos^48^, além do maior consumo desses dispositivos entre adultos jovens^45^.

Dessa forma, o aumento da prevalência do tabagismo identificado neste estudo pode estar relacionado não apenas às mudanças comportamentais provocadas pela pandemia, mas também à introdução e popularização desses novos produtos no país^45^ ^,^ ^48^. A ausência de questões específicas sobre experimentação e uso desses dispositivos durante a pandemia limita a análise mais aprofundada dessa hipótese.

O estudo aborda um tema de grande relevância em saúde pública e alerta para possíveis retrocessos nas políticas nacionais de controle do tabaco, bem como dificuldades para atingir as metas de redução do tabagismo^49^. Destaca-se a forte atuação da indústria do tabaco nas mídias sociais, com conteúdos enganosos e pressão para liberação de um novo marco regulatório que inclui a liberação de produtos como cigarros eletrônicos (vapes)^50^, o que pode influenciar negativamente o uso de tabaco no país. Assim, é crucial fortalecer ações de promoção da saúde, educação e regulação, em alinhamento com a Convenção-Quadro para o Controle do Tabaco da Organização Mundial da Saúde (OMS)^51^. Também é necessário planejar medidas específicas para assegurar a continuidade dessas políticas em contextos de crise, como catástrofes e pandemias.

Este estudo avança ao investigar o comportamento do uso de cigarros no Brasil antes, durante e após a pandemia, analisando fatores de iniciação e inovando com a amostragem RDS. Contudo, algumas limitações devem ser consideradas, especialmente aquelas inerentes às pesquisas por amostragem realizadas via internet. Pessoas sem acesso à rede têm probabilidade nula de serem selecionadas, enquanto indivíduos com baixa escolaridade e idosos frequentemente apresentam dificuldades para preencher o questionário on-line, o que pode interromper o desenvolvimento das redes de recrutamento. Além disso, como os participantes são voluntários, as probabilidades de seleção e as taxas de não resposta não podem ser estimadas, o que limita a inferência estatística. Outro desafio refere-se à obtenção de uma amostra representativa da população brasileira. No entanto, neste estudo, empregou-se um procedimento de pós-estratificação baseado nas estimativas populacionais da PNAD 2022, o que resultou em distribuições de variáveis muito semelhantes às observadas na PNAD. Entretanto, a exclusão de variáveis associadas aos desfechos analisados no processo de pós-estratificação pode introduzir vieses nas estimativas médias.

Os dados autorrelatados estão sujeitos a viés de memória e erro de informação; contudo, o uso de instrumentos padronizados fortalece as análises e permite a comparabilidade com outros estudos. Além disso, a prevalência de uso do tabaco na pandemia deve ser interpretada com cautela, pois não foi possível identificar quem já era fumante e deixou de fumar durante esse período.

O estudo mostra que a pandemia de COVID-19 intensificou o tabagismo no Brasil, com alta incidência durante o período e aumento da prevalência após seu término. A iniciação ao tabaco esteve associada a fatores individuais e contextuais, com maior vulnerabilidade entre jovens, pessoas sem companheiro(a), indivíduos com piora emocional e aqueles que aumentaram o consumo de bebidas alcóolicas. Esses resultados reforçam a necessidade de políticas voltadas a grupos vulneráveis, especialmente jovens e pessoas com piora da saúde mental, além de ações integradas de prevenção ao uso de álcool e tabaco.
